# Health and Development at Age 19–24 Months of 19 Children Who Were Born with Microcephaly and Laboratory Evidence of Congenital Zika Virus Infection During the 2015 Zika Virus Outbreak — Brazil, 2017

**DOI:** 10.15585/mmwr.mm6649a2

**Published:** 2017-12-15

**Authors:** Ashley Satterfield-Nash, Kim Kotzky, Jacob Allen, Jeanne Bertolli, Cynthia A. Moore, Isabela Ornelas Pereira, André Pessoa, Flavio Melo, Ana Carolina Faria e Silva Santelli, Coleen A. Boyle, Georgina Peacock

**Affiliations:** ^1^Oak Ridge Institute for Science and Education, Oak Ridge, Tennessee; ^2^Eagle Global Scientific, San Antonio, Texas; ^3^National Center on Birth Defects and Developmental Disabilities, CDC; ^4^Ministry of Health Brazil; ^5^Hospital Infantil Albert Sabin, Fortaleza, Ceará, Brazil; ^6^Hospital Regional de Guarabira/Governo do Estado da Paraíba, Paraíba, Brazil; ^7^Center for Global Health, CDC Brazil.

In November 2015, the Brazilian Ministry of Health (MOH) declared the Zika virus outbreak a public health emergency after an increase in microcephaly cases was reported in the northeast region of the country (*1*). During 2015–2016, 15 states in Brazil with laboratory-confirmed Zika virus transmission reported an increase in birth prevalence of microcephaly (2.8 cases per 10,000 live births), significantly exceeding prevalence in four states without confirmed transmission (0.6 per 10,000) (*2*). Although children with microcephaly and laboratory evidence of Zika virus infection have been described in early infancy (*3*), their subsequent health and development have not been well characterized, constraining planning for the care and support of these children and their families. The Brazilian MOH, the State Health Secretariat of Paraíba, and CDC collaborated on a follow-up investigation of the health and development of children in northeastern Brazil who were reported to national surveillance with microcephaly at birth. Nineteen children with microcephaly at birth and laboratory evidence of Zika virus infection were assessed through clinical evaluations, caregiver interviews, and review of medical records. At follow-up (ages 19–24 months), most of these children had severe motor impairment, seizure disorders, hearing and vision abnormalities, and sleep difficulties. Children with microcephaly and laboratory evidence of Zika virus infection have severe functional limitations and will require specialized care from clinicians and caregivers as they age.

The Zika Outcomes and Development in Infants and Children (ZODIAC) investigation sought to compile a comprehensive description of health and development among children aged >12 months who were born with microcephaly and participated in a 2016 case-control investigation. The case-control investigation assessed the association of Zika virus infection and microcephaly among children aged 1–7 months, living in Paraíba state. The children and their caregivers were evaluated by multidisciplinary teams at two state clinics in Campina Grande and João Pessoa (macroregions 1 and 2) in Paraíba state during August–October 2017. This report describes a subsample of 19 children, aged 19–24 months, who participated in ZODIAC and were born with microcephaly and with laboratory evidence of Zika virus infection.

All children in the ZODIAC investigation were born from October 1, 2015 through January 31, 2016, and were reported to the Registro de Eventos de Saúde Pública (RESP)—Microcefalias, Brazil’s national microcephaly registry. For infants to be eligible for the 2016 case-control investigation, their mothers must have resided in Paraíba state for at least 80% of their pregnancy. For the ZODIAC investigation, microcephaly was defined as head circumference below the third percentile for gestational age and sex, according to INTERGROWTH 21st standards (*4*). Subsequent measurements are reported in standard deviations (SD) to better characterize growth deficiencies (*5*). Laboratory evidence of Zika virus infection was defined as a positive test for Zika virus immunoglobulin M (IgM) and virus specific-neutralizing antibodies or a positive test for Zika virus-specific neutralizing antibodies in an infant sample (*6*). Samples were obtained at age 1–7 months in the 2016 case-control investigation, and any evidence of infection was assumed to be prenatal in origin. Results of prenatal and newborn testing to rule out other congenital infections were available for some infants and their mothers.

ZODIAC data were collected through clinical evaluations, caregiver interviews, and review of medical records. Licensed physicians performed growth, ophthalmologic and physical exams, and a neurologic assessment. Physicians were trained to use the Hammersmith Infant Neurological Examination (HINE), a standardized neurologic exam, to assess neuromotor function and visual and auditory responses (*7*). Trained interviewers administered screening and assessment instruments to the primary caregiver (usually the mother) regarding the child’s health and development, including a seizure screener (*8*), the Ages and Stages Questionnaires (ASQ-3),* and the Ages and Stages Social-Emotional Questionnaires (ASQ:SE).^†^ Data were captured in REDCap, a secure web application.

The families of 278 previously studied children residing in the ZODIAC investigation catchment area were eligible for inclusion; 122 children were enrolled, including 19 who were aged <24 months and who had both microcephaly at birth and laboratory evidence of Zika virus infection. Among the 19 children, 11 had a blood specimen that tested positive for Zika virus-specific IgM antibodies and neutralizing antibodies against Zika virus, and eight had only neutralizing antibodies against Zika virus. Among the eight with neutralizing antibodies only, seven had at least one test for other congenital infections; one had a positive Toxoplasma immunoglobulin G (IgG) antibody result and one had positive rubella virus and cytomegalovirus IgG results. Both had negative IgM antibody results for these infections; the first had brain imaging findings consistent with congenital Zika virus infection and the second had no record of imaging.

The median age at follow-up evaluation was 22 months (range = 19–24 months); 10 were male and nine were female. At the time of assessment, 15 children (seven males and eight females) had head circumference measurements more than 3 SDs below the mean for their age and sex (Table 1) (Table 2). Four children had an increase in head circumference for age from birth measurements: three males had head circumference within 1 SD below the mean and one female had head circumference within 1 SD above the mean. Thirteen children (six males and seven females) had length measurements 1–3 SDs below the mean, and 13 children (six males and seven females) had weight measurements 1 to >3 SDs below the mean for their age and sex.

**TABLE 1:** Growth measurements* of children aged 19–24 months with confirmed or probable congenital Zika virus infection^†,§^ and microcephaly classification at birth^¶,**^ — Paraíba, Brazil, August–October 2017

Growth	No. (%)	
	Male (n = 10)	Female (n = 9)
Head circumference^††^		
>3 SD below mean for age and sex^§§^	7 (70)	8 (89)
Length^¶¶^		
1–3 SD below mean for age and sex***	6 (60)	7 (78)
Weight^†††^		
1 to >3 SD below mean for age and sex^§§§^	6 (60)	7 (78)

**Abbreviation:** SD = standard deviation.

* http://www.who.int/childgrowth/standards/en.

^†^ Confirmed congenital Zika virus infection was indicated by a positive Zika virus-specific immunoglobulin M [IgM] capture enzyme-linked immunosorbent assay [MAC-ELISA] result on infant cerebrospinal fluid [CSF] or serum) and positive plaque reduction neutralization testing (PRNT). Serologic evidence without confirmation via PRNT indicated probable congenital Zika virus infection.

^§^ http://jcm.asm.org/content/38/5/1823.full.pdf+html.

^¶^ Microcephaly at birth was defined according to the internationally accepted definition, head circumference below the 3rd percentile for gestational age and sex, from the standards for newborns and references for very preterm infants compiled by the International Fetal and Newborn Growth Consortium for the 21st Century.

** https://intergrowth21.tghn.org/.

^††^ http://www.who.int/childgrowth/standards/hc_for_age/en/.

**^§§^** Of the remaining males, three (30%) had a head circumference equal to the mean or up to 1 SD below the mean, and of the remaining females, one (11%) had a head circumference equal to the mean or up to 1 SD above the mean.

^¶¶^ http://www.who.int/childgrowth/standards/height_for_age/en/.

*** Of the remaining males, the length of 4 (40%) was equal to the mean or up to 3 SDs above the mean, and of the remaining females, the length of 2 (22%) was equal to the mean or up to 1 SD above the mean.

^†††^ http://www.who.int/childgrowth/standards/weight_for_age/en/.

^§§§^ Of the remaining males, the weight of 3 (30%) was equal to the mean or up to 2 SDs above the mean; the weight of 1 (10%) male was >3 SDs above the mean. Of the remaining females, the weight of 2 (22%) was equal to the mean or up to 2 SDs above the mean.

**TABLE 2:** Growth parameters,* evaluations, and medical and developmental conditions for 19 infants aged 19–24 months with confirmed or probable congenital Zika virus infection,^†,§^ and microcephaly classification^¶,**^ at birth — ZODIAC investigation, Paraíba, Brazil, August–October 2017

Infant no.	Sex	Birth HC^**^ (%)	ZODIAC HC^††^ (Z score)	ZODIAC weight^§§^ (Z score)	Brain imaging consistent with CZS	Zika laboratory evidence	Seizures	Eating challenges	Sleep challenges	Severe motor impairment	Vision limitation	Hearing abnormalities	ASQ-3 age interval^¶¶^
1	F	<3rd	-7.85	-1.68	Yes	IgM +; NAb +	Yes	Yes	Yes	Yes	Yes	Yes	<6 months
2	F	<3rd	-7.21	-0.98	Yes	IgM +; NAb +	No	No	Yes	Yes	Yes	Yes	<6 months
3	F	<3rd	-7.08	-4.47	Yes	IgM +; NAb +	Yes	No	Yes	Yes	No	No	<6 months
4	M	<3rd	-4.88	-2.40	Yes	NAb + only	No	Yes	No	Yes	No	Yes	<6 months
5	M	<3rd	-4.20	1.90	Yes	NAb + only	Yes	No	Yes	Yes	Yes	Yes	<6 months
6	F	<3rd	-5.36	-0.86	Yes	IgM +; NAb +	No	No	No	Yes	No	Yes	<6 months
7	F	<3rd	-8.02	-1.56	Yes	NAb + only	Yes	Yes	No	Yes	Yes	No	<6 months
8	M	<3rd	-5.75	-4.11	Yes	IgM +; NAb +	Yes	No	No	Yes	No	Yes	<6 months
9	M	<3rd	-5.83	-1.46	Yes	IgM +; NAb +	No	Yes	No	Yes	Yes	Yes	<6 months
10	F	<3rd	-6.65	-1.23	Yes	IgM +; NAb +	Yes	Yes	Yes	Yes	Yes	Yes	<6 months
11	F	<3rd	-5.67	-0.91	Yes	NAb + only	Yes	Yes	No	Yes	Yes	Yes	<6 months
12	M	<3rd	-3.69	3.52	Yes	IgM +; NAb +	Yes	No	Yes	Yes	Yes	Yes	<6 months
13	M	<3rd	-7.03	-2.36	Yes	IgM +; NAb +	Yes	No	Yes	Yes	Yes	Yes	<6 months
14	F	<3rd	-8.45	0.18	Yes	IgM +; NAb +	Yes	Yes	No	Yes	Yes	Yes	<6 months
15	M	<3rd	-6.29	-1.60	Yes	IgM +; NAb +	Yes	Yes	No	Yes	Yes	Yes	<6 months
16	M	<3rd	-0.68	1.52	No record	NAb + only	No	No	Yes	No	No	No	>6 months
17	M	<3rd	-0.18	-0.87	No record	NAb + only	No	No	Yes	No	No	No	>6 months
18	F	<3rd	0.23	1.28	No anomaly	NAb + only	No	Yes	No	No	No	No	>6 months
19	M	<3rd	-0.09	1.14	No record	NAb + only	No	No	Yes	No	No	No	>6 months

**Abbreviations:** ASQ-3 = Ages and Stages-III Questionnaire; CZS = congenital Zika syndrome; F = female; HC = head circumference; IgM = immunoglobulin M; M = male; NAb = neutralizing antibodies; ZODIAC = Zika Outcomes and Development in Infants and Children.

* http://www.who.int/childgrowth/standards/en.

^†^ Confirmed congenital Zika virus infection was indicated by a positive Zika virus-specific IgM capture enzyme-linked immunosorbent assay result on infant cerebrospinal fluid or serum) and positive plaque reduction neutralization testing (PRNT). Serologic evidence without confirmation via PRNT indicated probable congenital Zika virus infection.

^§^ http://jcm.asm.org/content/38/5/1823.full.pdf+html.

^¶^ Microcephaly at birth was defined according to the internationally accepted definition, head circumference below the 3rd percentile for gestational age and sex from the standards for newborns and references for very preterm infants compiled by the International Fetal and Newborn Growth Consortium for the 21st Century.

** https://intergrowth21.tghn.org/.

^††^ http://www.who.int/childgrowth/standards/hc_for_age/en/.

^§§^ http://www.who.int/childgrowth/standards/weight_for_age/en/.

^¶¶^ The ASQ-3 is a series of 21 parent-completed questionnaires designed to screen the developmental performance of children aged 1–66 months in the areas of communication, gross motor skills, fine motor skills, problem solving, and personal-social skills (http://agesandstages.com); based on ASQ-3 screening, an age interval of <6 months indicates that the child’s parent-reported developmental progress has not advanced beyond that typical of an infant at age 6 months.

Eleven children screened positive for nonfebrile seizures, indicating possible seizure disorder (Table 2) (Table 3). Caregivers reported that eight children were previously hospitalized, including six hospitalized for bronchitis/pneumonia, and that 10 children had frequent sleeping difficulties and nine had eating or swallowing challenges. Thirteen children had an impaired response to auditory stimuli. Four children had retinal abnormalities and 11 had an impaired response to visual stimuli. Fifteen children did not pass the ASQ-3 age interval questionnaire designed for a child aged 6 months. Fifteen children had a global score below 40 on the HINE, indicating severe motor impairment, including 14 who had findings consistent with cerebral palsy (*7*). Outcomes including feeding challenges, sleeping difficulties, severe motor impairment, vision and hearing abnormalities, and seizures tended to co-occur. All children had at least one of these outcomes, 12 had three to five of these outcomes, and two had all six outcomes. Four children (infant number 16, 17, 18, and 19) (Table 2) had typical growth and development at follow-up and might have been misclassified at birth.

**TABLE 3:** Health and developmental outcomes of 19 children aged 19–24 months with confirmed or probable congenital Zika virus infection,*^,†^ and microcephaly classification^§,¶^ at birth — Paraíba, Brazil, August–October 2017

Outcome	No. (%)
**Medical findings**	
Seizures**^,††^	11 (58)
Retinal abnormalities^§§^	4 (21)
Hospitalization**	8 (42)
Pneumonia/Bronchitis	6 (75)
Intestinal infection	1 (14)
High fever	1 (14)
Failure to thrive/feed	1 (14)
**Functional outcomes**	
Sleeping difficulties**	10 (53)
Feeding difficulties**	9 (47)
Impaired response to auditory stimuli (hearing asymmetric or no response)^¶¶^	13 (68)
Impaired response to visual stimuli^¶¶^	11 (58)
**Neurologic outcomes^¶¶^**	
Severe motor impairment^¶¶^	15 (79)
Cerebral palsy***	14 (74)

* Confirmed congenital Zika virus infection was indicated by a positive Zika virus-specific immunoglobulin M capture enzyme-linked immunosorbent assay result on infant cerebrospinal fluid or serum and positive plaque reduction neutralization testing (PRNT) at birth. Serologic evidence without confirmation via PRNT indicated probable congenital Zika virus infection.

^†^ http://jcm.asm.org/content/38/5/1823.full.pdf+html.

^§^ Microcephaly at birth was defined according to the internationally accepted definition, head circumference below the 3rd percentile for gestational age and sex from the standards for newborns and references for very preterm infants compiled by the International Fetal and Newborn Growth Consortium for the 21st Century.

^¶^ https://intergrowth21.tghn.org/.

** Reported by the caregiver.

^††^ https://doi.org/10.1016/j.pediatrneurol.2015.09.016.

^§§^ Retinal abnormalities were identified by ophthalmologic exam.

^¶¶^ Motor function, functional hearing, and functional vision were assessed using the Hammersmith Infant Neurologic Exam (HINE). A global score below 40 on the HINE is associated with severe motor impairment, according to findings published in 2016 (https://doi.org/10.1111/dmcn.12876).

*** Cerebral palsy was identified by neurologist.

## Discussion

As of September 2017, 2,986 newborns with microcephaly in Brazil were reported to RESP and 2,959 cases are being monitored (*9*). Children with Zika virus–associated microcephaly face medical and functional challenges that span many areas of development. Previous reports established a baseline of poor health outcomes at birth, including severe brain and ophthalmologic abnormalities, and other serious central nervous system abnormalities (*3*). This report expands on initial findings by demonstrating that specific outcomes, such as severe motor impairment and impaired visual and auditory response to stimuli, affect the majority of children with evidence of congenital Zika virus infection and microcephaly and become more apparent as these children age. Approximately three quarters of young children affected by Zika virus infection in this analysis had at least three of the specified co-occurring outcomes. Many of the initial findings identified at birth remain present at ages 19–24 months, and these children are falling far behind in achievement of age-appropriate developmental milestones, indicating the need for long-term follow-up and support.

The findings in this report are subject to at least four limitations. First, although all children with microcephaly recruited into the 2016 case-control investigation from selected areas of Paraíba state were offered enrollment in the ZODIAC investigation, not all families chose to participate. Consequently, the findings might not be representative of all children with microcephaly associated with congenital Zika virus infection. Second, errors in head circumference measurement at birth and passive transfer of maternal antibodies might have led to misidentification and might explain the divergent observations for the four children showing more typical development. Additionally, some of the parent-assessment findings, such as those from the seizure screener, were not medically verified. Finally, the ages of infants in the original case-control investigation ranged from 1 to 7 months at the time of blood collection, and it is possible that the laboratory results for some infants reflected postnatal, rather than prenatal, exposure.

This report provides information on the ongoing challenges facing children with severe congenital Zika virus syndrome; these children will require specialized care from clinicians and caregivers as they age. These findings allow for anticipation of medical and social service needs of affected children and their families, including early intervention services, and planning for resources to support these families in health care and community settings in Brazil, the United States, and other countries. Children with disabilities related to congenital Zika virus infection will need multidisciplinary care from various pediatric subspecialists (*10*). Long-term follow-up and measurement of developmental progression of children affected by Zika virus can inform intervention services and sub-specialties needed to provide optimal care for these children.

**Summary**

**What is already known about this topic?**

Congenital Zika virus infection has been linked to increased rates of microcephaly and a unique pattern of birth defects among infants. Although children with microcephaly and laboratory evidence of Zika virus infection have been described in early infancy, the subsequent health and development in young children have not been well characterized, constraining planning for the care of these children.

**What is added by this report?**

The growth and development of 19 children, aged 19–24 months, with laboratory evidence of Zika virus infection were thoroughly assessed. All children had at least one adverse outcome including feeding challenges, sleeping difficulties, severe motor impairment, vision and hearing abnormalities, and seizures, and these outcomes tended to co-occur.

**What are the implications for public health practice?**

Children with microcephaly and laboratory evidence of Zika virus infection face medical and functional challenges that span many areas of development, some of which become more evident as children age. They will continue to require specialized care from clinicians and caregivers. These data allow for anticipation of medical and social services needs of affected children and families, such as early intervention services, and planning for resources to support these families in healthcare and community settings.

## Footnotes

* A series of 21 parent-completed questionnaires designed to screen the developmental performance of children aged 1–66 months in the areas of communication, gross motor skills, fine motor skills, problem solving, and personal-social skills. The age-appropriate questionnaire is completed by the parent or caregiver (http://agesandstages.com).

† A series of nine age-appropriate parent-completed questionnaires designed to screen young children for social-emotional issues during the first 6 years of life for seven social-emotional areas: self-regulation, compliance, adaptive functioning, autonomy, affect, social-communication, and interaction with people (http://agesandstages.com).
